# How teachers’ caring behavior impacts the socio-cultural adaptability of international students: a chain mediation model

**DOI:** 10.3389/fpsyg.2025.1656033

**Published:** 2025-10-10

**Authors:** Zilong Yin, Yuhao Zhao, Yuefang Wang

**Affiliations:** 1 School of Teacher Education, Jiangsu University, Zhenjiang, Jiangsu, China; 2 School of Psychology and Educational Sciences, Zaozhuang University, Zaozhuang, Shandong, China

**Keywords:** teachers’ caring behavior, socio-cultural adaptability, perceived social support, resilience, international students

## Abstract

Enhancing socio-cultural adaptability (SCA) is critical to improving the educational quality of international students. Guided by cultural adaptation theory, this study tested whether teachers’ caring behavior (TCB) promotes SCA in online education and examined the mediating roles of perceived social support (PSS) and resilience (RS). A sample of international students (*N* = 872) completed validated scales for TCB, PSS, RS, and SCA; data were analyzed with SPSS 27.0 and AMOS 21.0 using stratified linear regression and mediation models. Results showed that TCB significantly and positively predicted SCA, RS partially mediated the TCB–SCA association, and PSS affected the TCB–SCA relationship only indirectly through RS. These findings clarify the interplay among TCB, PSS, RS, and SCA in online settings and provide theoretical and practical guidance for interventions that enhance international students’ SCA by strengthening resilience and supportive environments.

## 1 Introduction

In the 2020–2021 academic year, amid the global disruption caused by the COVID-19 pandemic, the international students registered in China came from 195 countries and regions, with 76% of them pursuing academic degrees—a significant increase of 35 percentage points compared to 2012 (Ministry of Education of PRC, 2022). As the world’s largest source country for international students and the leading destination for higher education in Asia, China has demonstrated vast potential in developing its “Study Abroad in China” initiative. In line with this trend, the Ministry of Education issued a policy in 2020 emphasizing the need to strengthen the “Study Abroad in China” brand and improve the quality and management of education and training for international students (Ministry of Education of PRC, 2020). Enhancing the global competitiveness of China’s higher education system relies not only on institutional reforms but also on the engagement of teachers and the socio-cultural adaptability (SCA) of international students. Prior research has shown that SCA plays a crucial role in fostering international students’ personal development and academic achievement in cross-cultural learning environments (Fong, 2020; Shadiev et al., 2021).

Cultural adaptation theory, rooted in behavioral science, provides a foundational framework to explore how individuals adapt to new cultural contexts. It defines SCA as comprising both cultural and psychological adaptation, underpinned by mechanisms such as cultural learning and stress-coping processes (Fanari, 2025). Communication is central to this adaptation process, and teacher–student interactions are recognized as vital to international students’ behavioral adjustment in unfamiliar cultural environments. Cross-cultural adaptation is influenced by a range of variables that evolve over time, with cultural learning experiences emerging as a major predictor (Ward, 2001; Searle and Ward, 1990). For international students, the pressures of adjusting to different academic systems, educational norms, and cultural values can lead to heightened stress, affecting both mental health and subjective well-being (Bethel et al., 2020). Empirical findings from Turkey further confirm a strong positive correlation between SCA and psychological adaptation (Gibbs et al., 2020).

In the Chinese context, factors such as students’ learning styles and psychological states (Li et al., 2023), teacher–student relationships (Liu, 2025), and the buffering role of social support in enhancing well-being (Bethel et al., 2020) have all been shown to influence SCA. According to self-determination theory, individuals are more likely to regulate their behaviors autonomously when motivated by intrinsic values and personal goals (Yang et al., 2023). Furthermore, studies have established that perceived social support (PSS) and resilience (RS) can alleviate stress and improve psychological outcomes, thereby facilitating better adaptation (Tang, 2024; Almukdad, 2024; Tol et al., 2013). Teachers, as primary figures in students’ academic and emotional lives, play a significant role in this process. Teachers’ caring behavior (TCB)—reflected through emotional, instructional, and moral support—has been shown to reduce anxiety and enhance learning outcomes (Hou, 2024; Sutton and Wheatley, 2003). Moreover, educational interactions can foster cross-cultural competence (Shadiev et al., 2021), while strong teacher–student relationships offer critical social support that enhances international students’ SCA (Cifuentes-Faura et al., 2021). However, despite these insights, few studies have examined how teachers’ caring behavior influences socio-cultural adaptability in online contexts using a chain mediation model, especially in Eastern cultural settings (Yu et al., 2019).

Therefore, this study integrates the cultural adaptation theory from Ward et al. with self-determination theory to investigate the mechanism through which TCB impacts international students’ SCA. Specifically, it explores the mediating roles of PSS and RS, aiming to provide empirical evidence and practical guidance for educators to enhance the adaptive experiences of international students in digital learning environments. This research also contributes to practical applications in academia and student support, providing insights into meeting the unique needs of international students in educational environments. Grounded in cross-cultural adaptation theory and self-determination theory, the proposed model establishes theoretical foundations for the hypothesized relationships. Cross-cultural adaptation theory posits that individuals experience behavioral and psychological adjustments when adapting to a new cultural environment, emphasizing the importance of cultural learning, stress-coping, and interpersonal interactions (Ward, 2001; Fanari, 2025). Teachers’ caring behavior (TCB) serves as a key interpersonal factor that facilitates international students’ adaptation by providing emotional and instructional support, thereby reducing acculturative stress and enhancing socio-cultural adaptability (H1). Furthermore, TCB enhances perceived social support (PSS), a vital external resource in adaptation processes (H2), and fosters resilience (RS), a personal trait that supports coping with cultural stressors (H3). Self-determination theory further explains how TCB satisfies students’ basic psychological needs for autonomy, competence, and relatedness (Ryan and Deci, 2000), thereby promoting internal motivation that contributes to greater resilience and stronger perceptions of support. By integrating both theories, this study proposes a chain mediation mechanism (H4) in which PSS and RS sequentially mediate the relationship between TCB and socio-cultural adaptability, offering a comprehensive framework to understand how external care translates into internal adjustment capacities.

## 2 Literature review

### 2.1 SCA and TCB

SCA refers to the ability to adapt to the local socio-cultural environment and whether individuals can effectively communicate with the local people (Ward and Kennedy, 1992). Ward and his colleagues distinguish SCA from psychological adaptation (Fanari, 2025), and this distinction has been widely recognized in contemporary cultural adaptation research (Khazhgaliyeva, 2023). It integrates knowledge and skills, including not only proficiency in basic communication and social interaction skills but also adaptation to new environments, norms, values, and worldviews, emphasizing the pivotal role of communication in cross-cultural encounters (Fanari, 2025). Sam and Berry (2010) also agree with the importance of communication, stating that whether cross-cultural learners can achieve good cross-cultural communication mainly depends on how individuals change themselves, and divides cultural adaptation into four levels according to the degree to which they can change themselves, namely integration, assimilation, separation, and marginalization. Bhawuk (1998) believed that the opportunity to learn and acquire specific cultural knowledge is a prerequisite for SCA. Due to the fact that SCA refers to behavioral skills and is set within the framework of cultural learning, research on its antecedents or predictive factors mainly focuses on contextual variables related to the learning process, such as general knowledge about a new culture (Fanari, 2025). As cultural learning is largely a form of social learning, the role of cross-cultural interaction in the process of SCA is also emphasized (Searle and Ward, 1990).

In Bronfenbrenner’s “ecosystem theory,” schools act as vital living and learning contexts for international students, providing crucial social support beyond their families (Zhang, 2025). The main and most typical cross-cultural interactions happen in school settings for international students, namely interactions with Chinese teachers and international students, a largely overlooked area of SCA influence.

TCB refers to the many teacher activities aimed at building good teacher-student relationships, ensuring the expected education results, fulfilling teaching tasks with due diligence in the education or teaching process, investing sufficient time and energy to support students development, and providing a tolerant attitude toward students (Hou, 2024). These behaviors include encouraging students to express their own opinions, providing rich extracurricular course selection content, treating each student with patience and respect, and other interactions during teaching. Teachers’ inclusive professional attitude plays an important role in the educational process and teacher-student interactions (Barnová et al., 2022). The quality of a teacher is one of the most essential environmental factors that affect the growth of young people in schools as they can help students actively cope with various challenges and adapt to society better (Sutton and Wheatley, 2003). According to the social learning theory of Albert Bandura, students learn relevant interpersonal relationships and conflict resolution strategies by observing teachers’ behavior and consequences. What’s more, successful teachers will not only enable students to achieve greater academic achievement but also help them learn better emotional reactions and enjoy a more positive classroom atmosphere (Goodenow, 1993; Teven, 2007). Some research results indicate that the reciprocal and guided discovery teaching style can help students master knowledge, enhance adaptive cognition, and achieve emotional reflection (Chaliawala, 2025). Also, students can gain more confidence and interest in a subject based on teacher skill. Indicating that teachers’ behavior is related to students’ performance (Gabrhelová and Čepelová, 2022).

Participation, interaction, and mutual assistance is precisely what TCB emphasizes (Zhang, 2024) and shows that TCB is a factor that may affect the adaptability of international students. Due to the impact of the COVID-19 pandemic, international students around the world have been attending online classes for nearly 3 years. Although the Chinese government’s policy changes on the COVID-19 pandemic at the end of 2022 revitalized the education of international students, online teaching as a gradually familiar teaching method had penetrated into the hearts of teachers and students. Online teaching relies on a degree of digitization, including the digital equipment of universities and the degree of teachers’ digitization level (Soekamto et al., 2022). There are currently only a few empirical studies on the TCB of online instruction. The first aim of the current study is to verify the relationship between TCB and the SCA of international students and to prove the effect of on-line teaching activities for SCA of international students. Combined with the previous findings and theoretical basis above, we hypothesized that:

*H1*: TCB is positively associated with the SCA of international students.

### 2.2 The mediating role of PSS

PSS refers to the subjective perception and evaluation individuals feel when they are supported, understood, and respected by the outside world (Sarason et al., 1991). Discussed in a large deal of literature in the form of mediation or moderator variables, social support plays a vital role in psychology, education, clinical medicine, and other fields (Tang, 2024; Wilcox et al., 2005). The existing research results show that PSS has a directly negative predictive effect on social anxiety (Lyu C. 2024; Lyu Y. 2024). Steese et al. (2006) found that PSS can protect college students from psychological distress and plays a role in buffering pressure. Recent research has also shed light on how adaptability is significantly related to PSS (Burns et al., 2018). When an individual arrives in a new environment, social support can stabilize the individual and enhance the relationship between the individual and SCA (Jyoti and Kour, 2017). At the same time, Chavajay (2013) illuminated that social support from the host culture plays a vital role in helping international students adjust their lives and study in new cultural context. Research has found that international students who lack PSS have the worst adaptability and tend to be marginalized (Holliman et al., 2021). Still, TCB allows students to perceive the positive attention of the teacher toward themselves, generating an emotional experience of being respected, supported, and understood; in other words, TCB can affect students’ PSS (Almukdad, 2024). Teachers act as “important others” for international students in the school environment, and the self-determination theory suggests that important others influence people’s motivation and behavior (He, 2024). When important others hold a caring and encouraging attitude toward an individual’s behavior, the individual can fully tap into their inner potential and actively improve and develop themselves (Ye et al., 2017). Consequently, due to the positive influence of TCB, international students are likely to consider teachers as a source of social support and positively associated with their SCA. In summary, the research review fully demonstrates the pivotal role of PSS in SCA, yet more validation is required to ascertain whether PSS can mediate the relationship between TCB and SCA. Accordingly, the following hypothesis is put forward that:

*H2*: The PSS plays a mediating role in the influence of TCB toward the SCA of international students.

### 2.3 The mediating role of RS

RS is the effective response and adaptation of individuals facing loss, difficulty, or adversity and is related to the interaction between their internal forces and external support factors obtained from the social environment. It is generally considered a protective factor to decrease the level of distress (Gundogan, 2021). In this study, more attention is paid to the qualitative definition of RS, which is regarded as a characteristic of an individual (Yu, 2024).

Self-determination theory proposes that once individual comprehend their own needs and environmental information, individual behavior becomes autonomous, accentuating a proactive personal role (Yang et al., 2023). When international students recognize cross-cultural environmental information and the need to adapt to the environment, they will modify their RS because of self-protection to help themselves adapt to the environment and overcome the difficulties brought about by cross-cultural communication (Tan et al., 2021; Gabrielli et al., 2022).

Although researchers have clearly shown a direct connection between RS and cultural adaptability (Tol et al., 2013), the exact mechanism by which RS affects international students’ SCA in school settings is still unclear. International students face more daily challenges due to cross-cultural environments, minor yet persistent. The daily interactions between teachers and international students, such as meticulous attention to life and learning, support, and respect for their perspectives, all have a significant impact on their happiness, and there may be significance to defining the basic rules of the school’s routine, potentially impacting the RS of international students (Dong et al., 2024). Previous studies clarified that RS is a crucial intermediary factor of SCA, which is associated with reduced psychological pressure, and also individuals with strong RS can adjust their own state and cognition more actively and effectively to cope with sudden changes in the environment and setbacks, thereby improving their SCA (Liao et al., 2019).

Furthermore, in the process of school education through interaction with teachers, students can maintain a positive attitude when facing difficulties if they can obtain satisfactory emotional support from teachers, thus learning effectively (Larcombe, 2024). These caring behaviors help students resist adversity and setbacks and overcome difficulties quickly, which benefits RS. Students with high RS actively seek teacher support and care and are more likely to successfully overcome school-related pressures, maintain optimal motivation levels to enhance their participation in learning, and achieve high performance, including exceptional grades and adaptation (Romano et al., 2021). We suggest exploring whether RS plays a mediating role in the relationship between TCB and the SCA of international students, and the following hypothesis is put forward that:

*H3*: The RS plays a mediating role in the influence of TCB toward SCA of international students.

### 2.4 The chain mediating role of PSS and RS

It is worth mentioning that good teacher-student interactions can make international students feel more social support (Wilcox et al., 2005). This kind of social support can act as an external protective factor for RS, helping international students alleviate the negative impact of the cross-cultural context on RS (Wilks, 2008). Empirical studies have also shown that PSS can positively predict RS (Sippel et al., 2015), and RS is also an important predictor of individual SCA (Tol et al., 2013). Thus, it is reasonable to assume that PSS and RS are mediating variables between TCB and SCA. However, although these two variables have been included in cross-cultural studies (Khawaja et al., 2017; Wang and Zhang, 2020), the chain mediating role of PSS and RS remain unexplored among international students in the framework of cross-cultural adaptation theory. Based on the above empirical findings and theory, we hypothesized that:

*H4*: PSS and RS act as chain mediators in the relationship between TCB and SCA of international students.

### 2.5 The present study

**Figure 1:**
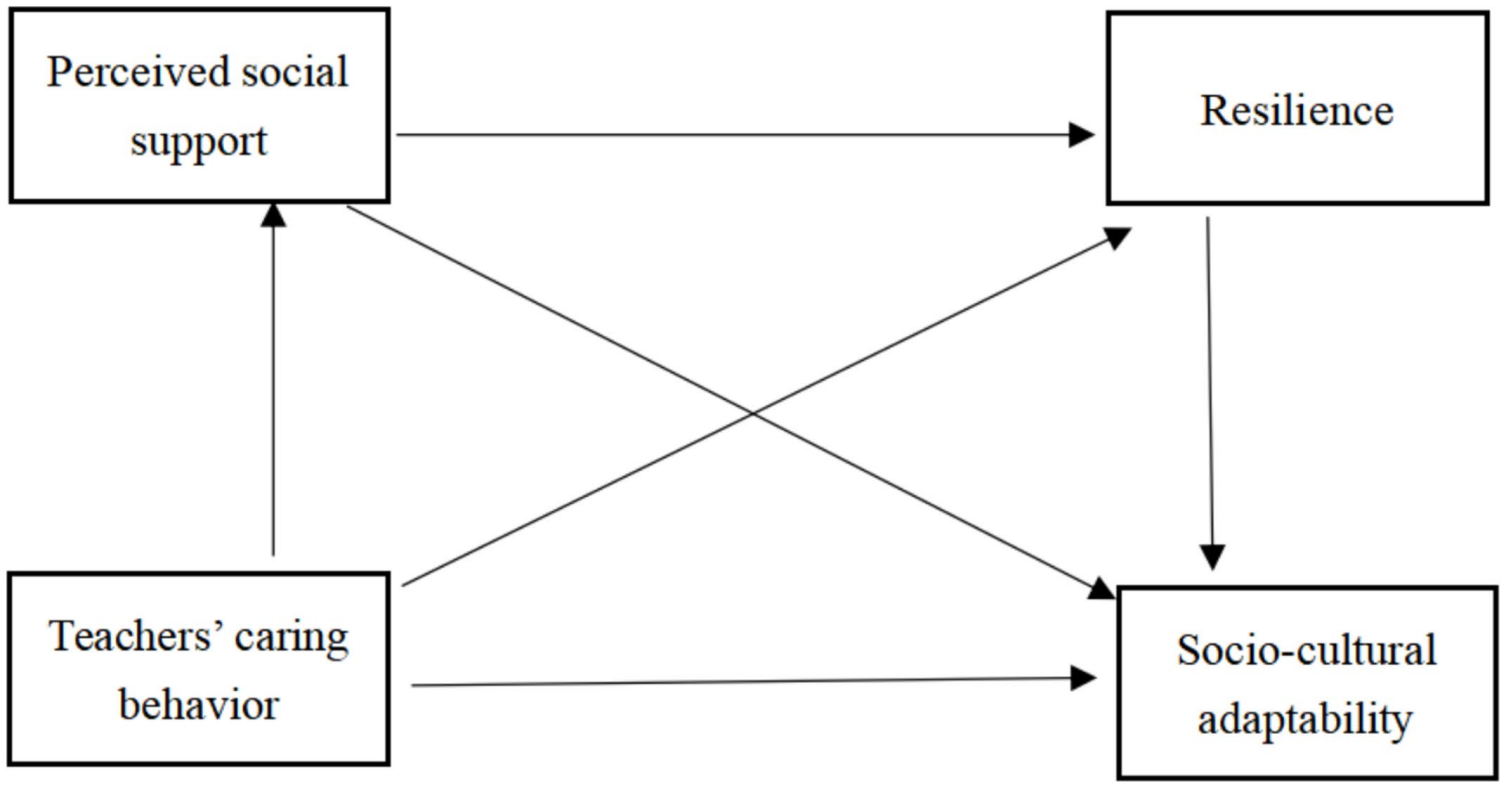
Conceptual mediation model.

The present study examined the relationship between TCB and the SCA and possible mediating factors among international students studying in China. However, there is no model that directly describes the relationship between the two variables. Based on the cross-cultural adaptation theory (Berry et al., 2006; Sam and Berry, 2010) and previous literature (e.g., Chavajay, 2013; Khawaja et al., 2017; Fanari, 2025; Larcombe, 2024; Sippel et al., 2015; Steese et al., 2006; Teven, 2007), we constructed a chain mediation model to test our hypotheses (see Figure 1). This model assumed that TCB is an independent variable, SCA is the dependent variable, and the mediators are the PSS and RS of international students studying in China. This study attempts to explain the relationship between them and reflect on incorporating these relationships into models that can be applied in the future as well as into some feasible intervention strategies. The aim is to provide support for international students to cope with their cross-cultural adaptation problems and promote their mental health and academic development.

## 3 Methods

### 3.1 Participants and procedure

The study was conducted at five comprehensive universities in the Jiangsu, Zhejiang, and Shanghai regions of China. A total of 994 international students filled out the scales, and 872 questionnaires remained after deleting invalid ones (incomplete responses, blank questionnaires, etc.), and the effective response rate was 87.73%. The participants (*N* = 872) were aged between 18 and 33 years (*M* = 23.14, SD = 3.21), and 54.2% were female. They came from 38 countries, with the majority originating from Asia (48.8%), Africa (28.5%), and Europe (12.6%). Regarding learning formats, 62.5% reported engaging in fully online learning, while 37.5% participated in a hybrid mode combining online and in-person courses. To assess the constructs of this study, we employed well-established and reliable instruments. Table 1 summarizes the measurement scales, their dimensions, item counts, and reliability coefficients (Cronbach’s α). Stratified regression analysis was conducted to examine the mediation effects while controlling for age, gender, student category, region of origin, and learning format. Furthermore, to address potential common method bias, Harman’s single-factor test was performed. The first factor accounted for only 26.7% of the total variance, suggesting that common method variance was not a serious concern in this study.

**Table 1:** Summary of measurement instruments, dimensions, item counts, and reliability.

Scale	Dimensions	No. of items	Cronbach’s α
Teachers’ Caring Behavior Scale (TCBS)	Clarity, inclusiveness, participation, accessibility	17	0.908
Perceived Social Support Scale (PSSS)	Family support, friend support, other support	12	0.833
Connor–Davidson Resilience Scale (CD-RISC)	Tenacity, self-improvement, optimism	25	0.916
Socio-Cultural Adaptability Scale (SCAS)	Cultural adaptation, psychological adaptation	29	0.945

The participants came from various countries in Asia, Europe, America, and Africa, having enrolled in Chinese universities before 2021 and continuing their studies into the spring semester of 2023. Following the adoption of the open-door policy by the Chinese government in early 2023 in response to the COVID-19 pandemic, international students who were quarantined outside of China were allowed to decide when to return to their universities based on their individual circumstances (e.g., financial situation, study duration, etc.). During the transitional period from fully online to offline teaching, Chinese universities continued to offer online instruction, and international students residing in China could also opt for online learning due to factors such as internet access or classroom composition. All international students who participated in this study chose to engage in online learning for all or part of their courses. Contact teachers at each university, responsible for managing international student affairs, distributed the questionnaire link and explained the purpose of the study. All participants voluntarily completed the online survey and were informed of their right to withdraw at any point. Confidentiality of the data was strictly maintained. Among the 872 participants (male = 397, 45.5%), there were four categories of international students: 151 (17.3%) were preparatory students, 304 (34.9%) were undergraduates, 241 (27.6%) were master’s students, and 176 (20.2%) were doctoral students. This constitutes a convenience sample suitable for statistical analysis (Putri, 2024).

### 3.2 Measures

#### 3.2.1 Teachers’ caring behavior scale

Li et al. (2021) developed the teachers’ caring behavior scale (TCBS) in Online Teaching on the basis of the previous scale; the Cronbach’s alpha of the entire scale is 0.952. The 17-item scale is composed of four parts: clarity (0.880), inclusiveness (0.902), participation (0.883) and accessibility (0.890). Some items of the scale include “Encourage and allow students to share different opinions or experiences from the teacher” and “Provide rich elective content online to facilitate students to expand on their own.” Participants gave their answers on a 5-point Likert scale ranging from 1 (not applicable to me) to 5 (very applicable to me). In the present study, the retest Cronbach’s alpha is 0.908, and the 4-factor load coefficients are 0.768, 0.853, 0.812, and 0.844, indicating that the scale is suitable for international students studying in China and also suitable for TCB in online and offline mixed teaching. Wu et al. (2024) validated the reliability of this scale.

#### 3.2.2 Perceived social support scale

The perceived social support scale (PSSS) used in this study is developed by Dahlem et al. (1991). The scale has 12 items in total and consists of three subscales: family support (relatives), friend support (classmates), and other support (teachers). Some items of the scale include “There is a special person who is around when I am in need” and “I can talk about my problems with my friends.” Participants answered on a 5-point Likert scale where 1 indicated “Did not apply to me at all” and 5 indicated “Applied to me very much.” The Cronbach’s alpha of the full scale and the three subscales are 0.91, 0.90, 0.94, and 0.95, respectively. In this scale, the main change is from “leaders, relatives, and colleagues” to “teachers, relatives, and classmates.” Such adjustments have shown reliable results, as shown in previous studies (Hefner and Eisenberg, 2009). The reset Cronbach’s alpha by perceived social support sources is full scale, 0.833. The result indicates that this scale is suitable for measuring the SCA of international students in China.

#### 3.2.3 Connor-Davidson resilience scale

In this study, RS was measured using the Connor-Davidson resilience scale (CD-RISC) (Wong, 2024); the scale consists of 25 items divided into three sub-factors: tenacity, self-improvement, and optimism. Some items of the scale include “Able to adapt to change” and “Pride in your achievements.” Answers were given on a 5-point Likert scale, where 1 indicated “Never” and 5 indicated “Always.” The Cronbach’s alpha of the full scale and the three subscales are 0.91, 0.88, 0.88, and 0.60, respectively. According to the realities of the respondents, the scale was slightly adjusted and the respondents were asked to answer questions according to the overall situation of the past month. The Cronbach’s alpha of the full scale in the present study is 0.916. Previous research has proved this scale’s robust reliability (Liu et al., 2020).

#### 3.2.4 Socio-cultural adaptability scale

This study adopted the socio-cultural adaptability scale (SCAS) compiled by Ward and Kennedy (1999) to measure the adaptability of international students. Specifically, they responded to 29 questions on a scale anchored from 1 (strongly disagree) to 5 (strongly agree), where high scores indicated high cultural adaptability. Some items of the scale include “Adapting to local accommodation” and “Being able to see two sides of an intercultural issue.” The Cronbach’s alpha of this scale is 0.75–0.91 (*M* = 0.85). In this study, the Cronbach’s alpha was 0.945, and the factor load coefficients of cultural adaptation and psychological adaptation were 0.792 and 0.814, the results, respectively, indicating that this scale is suitable for measuring the SCA of international students in China. Sam and Berry (2010) also demonstrated the high reliability of this scale.

### 3.3 Data analysis

Data analysis was performed with the SPSS Statistic Software version 27.0 and AMOS Software version 21.0. Based on a literature review, a hypothetical model was constructed. In this model, we considered the international students’ gender, age, family income, student category, learning style, and residence as control variables. The hypothesis is that TCB can directly predict the SCA of international students and the PSS and RS can serve as mediating variables to influence the relationship between TCB and SCA. Firstly, we conducted CFA testing on the model, and the results are shown in Table 2. According to Thompson’s (2004) suggestion, the fit of the model is acceptable. Based on this hypothetical model, correlation analysis was conducted on all variables in the study, and the results are shown in Table 3. According to the correlation results, the model fit was recalculated by adding three control variables (gender, student type, and learning style) to the existing model. It was found that χ^2^/*df* = 3.084, CFI = 0.959, RMSEA = 0.056, and the model fit was significantly better than before. Then, stratified regression tests were conducted on the influencing factors in the model to determine which factors have a greater impact on the SCA of international students during their cross-cultural learning process. Finally, the hypotheses of this study were validated by calculating the model path using Bootstrap in AMOS.

**Table 2:** Discriminant validity.

Model	Factors	χ^2^/*df*	RMSEA	CFI	GFI	IFI	TLI
4 factors	TCB/PSS/RS/SCA	3.638	0.076	0.946	0.923	0.952	0.945
3 factors	TCB+PSS/RS/SCA	10.174	0.151	0.914	0.905	0.905	0.911
2 factors	TCB+PSS+RS/SCA	23.114	0.204	0.798	0.687	0.798	0.750
1 factor	TCB+PSS+RS+SCA	28.560	0.241	0.723	0.652	0.752	0.520

TCB, Teachers’ Caring Behavior; PSS, Perceived Social Support; RS, Resilience; SCA, Socio-cultural Adaptability; *N* = 872.

**Table 3:** Correlation results.

Variable	*M* (SD)	1	2	3	4	5	6	7
1. Gender	1.54 (0.49)	1						
2. Student category	2.20 (1.09)	−0.270**	1					
3. Learning style	1.96 (0.81)	−0.090**	−0.032	1				
4. TCB	4.06 (0.78)	0.081*	−0.05	−0.014	1			
5. PSS	4.06 (0.77)	0.099**	−0.043	0.008	0.637**	1		
6. RS	4.08 (0.70)	0.024	0.112**	0.026	0.627**	0.653**	1	
7. SCA	4.09 (0.76)	−0.016	0.066	0.065	0.600**	0.570**	0.760**	1

TCB, Teachers’ Caring Behavior; PSS, Perceived Social Support; RS, Resilience; SCA, Socio-cultural Adaptability; *N* = 872; *p < 0.05; **p < 0.01.

## 4 Results

### 4.1 Confirmatory factor analysis test

This study used AMOS software and confirmatory factor analysis (CFA) to test the discriminant validity of the four variables in the research model, namely, TCB, PSS, RS, and SCA. Results in Table 2 demonstrate that the 4-factor model shows a good fit (χ^2^/df = 3.638, RMSEA = 0.076) and that the four variables have good discrimination validity, significantly better than that of the other three competition models. At the same time, the analysis results confirm that there is no obvious common method bias in the data of this study.

### 4.2 Pearson’s correlation analysis

In this study, SPSS27.0 was used to analyze the correlation of various research variables, and the results are shown in Table 3. At first, TCB, PSS, and RS were significantly positively correlated with the SCA of international students. TCB, PSS, and RS were also significantly positively correlated. In the end, there was a significant positive correlation between TCB and PSS. In the demography variable test of international students, it was found that students’ gender, student category, and learning style show various degrees of correlation with the independent and dependent variables, while age, residence, family income, and other factors do not show a correlation, so they are not shown in Table 3. Thus, the gender, student category, and learning style (online or mixed learning) of international students will be listed as control variables in the following research. To facilitate interpretation, Table 2 presents the descriptive statistics and Pearson correlation coefficients among the key variables. The results indicate that teachers’ caring behavior (TCB) is positively correlated with perceived social support (PSS, *r* = 0.58, *p* < 0.001), resilience (RS, *r* = 0.52, *p* < 0.001), and socio-cultural adaptability (SCA, *r* = 0.49, *p* < 0.001). These significant correlations support the hypotheses and lay the foundation for the mediation analysis.

### 4.3 Direct effects

In the study, SPSS was used to standardize all variables. Linear regression was then used to analyze the direct effects with all variables including control variables. The result is shown in Table 4. In Step 1, TCB significantly predicted SCA (β = 0.49, *p* < 0.001). In Step 2, the inclusion of PSS and RS as mediators reduced the direct effect of TCB on SCA (β = 0.18, *p* < 0.01), while both PSS (β = 0.28, *p* < 0.01) and RS (β = 0.32, *p* < 0.001) showed significant predictive effects, suggesting a partial mediation effect. While some of the control variables cannot significantly affect the SCA of international students in China, the regression results of student categories show that (β = 0.072, *p* < 0.001) they significantly and positively affected the RS of them. Meanwhile, the regression results of gender show that (β = 0.155, *p* < 0.001), which indicates a significant gender difference regarding PSS but no difference regarding SCA. TCB has a significant positive impact on the SCA of international students studying in China (β = 0.589, *p* < 0.001), so the H1 was supported. Moreover, through the calculation results of the linear regression, we know that TCB has a significant positive impact on RS (β = 0.691, *p* < 0.001) and PSS (β = 0.633, *p* < 0.001). The results also support the subsequent path analysis research of this study to verify further the hypotheses.

**Table 4:** Direct effect.

Independent variable (control variable)		Dependent variable	β	S. E.	r(Var.)
1. Gender	→	PSS	0.155***	0.086	0.099**
	→	RS	0.034	0.048	0.024
	→	SCA	−0.025	0.052	−0.016
2. Students category	→	PSS	−0.031	0.024	−0.043
	→	RS	0.072***	0.022	0.112**
	→	SCA	0.046	0.024	0.066
3. Learning style	→	PSS	0.008	0.033	0.008
	→	RS	0.022	0.030	0.026
	→	SCA	0.061	0.032	0.065
4. TCB	→	PSS	0.633***	0.026	0.637***
	→	RS	0.691***	0.029	0.627***
	→	SCA	0.589***	0.027	0.600**

TCB, Teachers’ Caring Behavior; PSS, Perceived Social Support; RS, Resilience; SCA, Socio-cultural Adaptability; *N* = 872; **p < 0.01; ***p < 0.001.

### 4.4 Indirect effects

This study utilized a structural equation model to test the mediating effect of PSS and RS on TCB and SCA. The present study used the Bootstrap method of repeated sampling 5,000 times in AMOS to calculate the mediating role of PSS and RS between TCB and SCA. According to the hypothesized model, the values of 4 paths within a 95% confidence interval were calculated to validate H2, H3, and H4. The specific results are shown in Table 5.

**Table 5:** Indirect effect.

Model pathways	Estimate	95%-confidence interval		*p*	S. E.
		Lower	Upper		
TCB → SCA	0.168	0.05	0.293	0.006	0.062
TCB → PSS → SCA	0.039	−0.008	0.087	0.105	0.024
TCB → RS → SCA	0.210	0.155	0.273	0.000	0.030
TCB → PSS → RS → SCA	0.161	0.125	0.207	0.000	0.020

TCB, Teachers’ Caring Behavior; PSS, Perceived Social Support; RS, Resilience; SCA, Socio-cultural Adaptability; *N* = 872.

**Figure 2:**
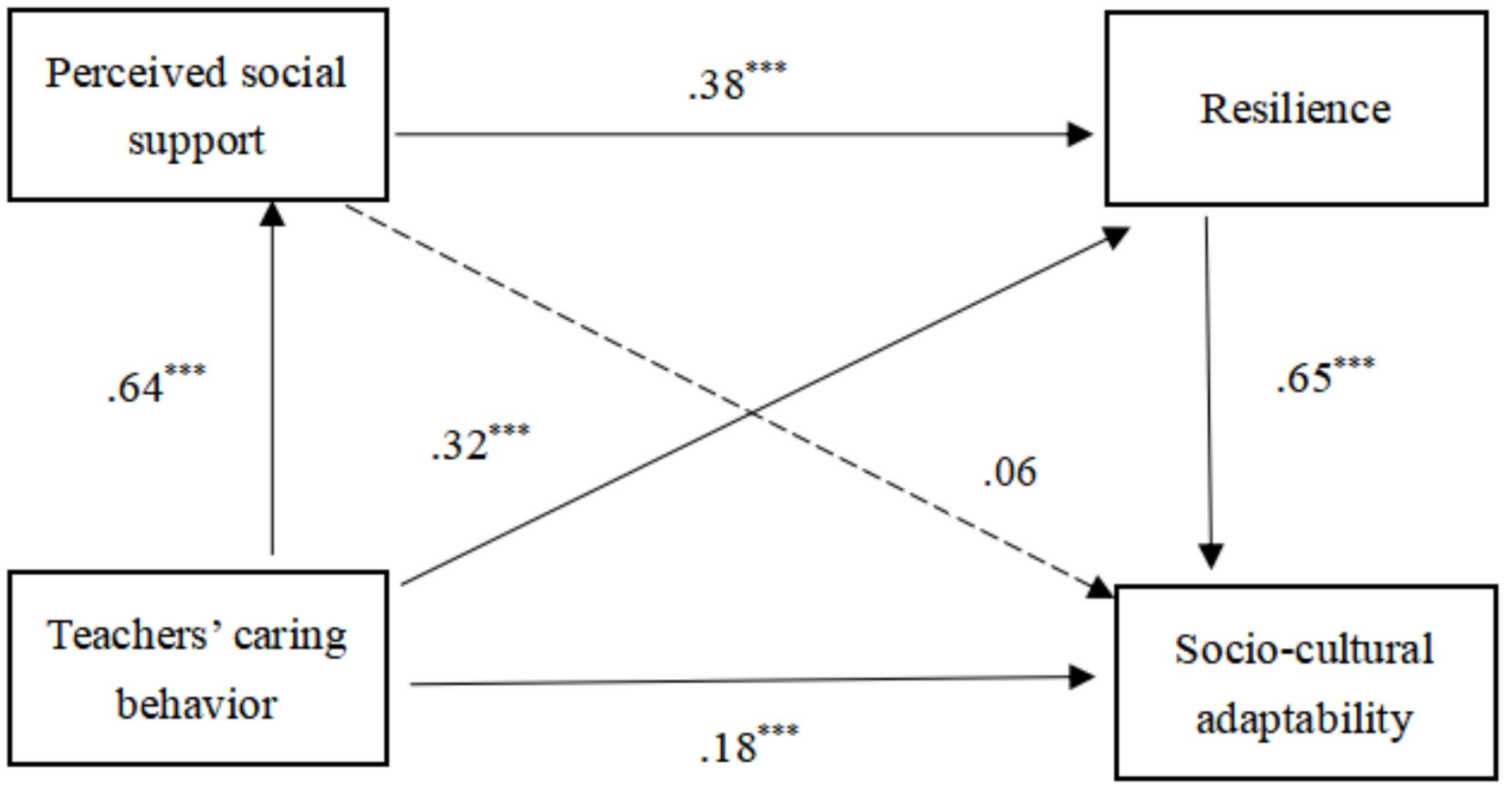
Results of the study model (***p < 0.001).

The results indicate that TCB cannot affect SCA through PSS, as the 95% confidence interval is [−0.008, 0.087], and the interval includes 0 (Parlak, 2025), *p* > 0.05, which means the mediating role effect is not tenable and H2 was not supported. However, TCB can significantly positively affect SCA through RS, as the 95% confidence interval is [0.155, 0.273], and the interval does not include 0 (*p* < 0.000), which means the independent mediating effect of RS is established and H3 was supported. Although TCB cannot affect SCA through PSS, PSS can have an impact on SCA through the mediation of RS, with a 95% confidence interval result of [0.125, 0.207], where the interval does not include 0 (*p* < 0.000). Therefore, the chain mediating effect of PSS and RS is established and H4 was supported. Meanwhile, the direct impact of TCB on SCA is 0.168 (*p* < 0.01), indicating that PSS and RS play a partial mediating role in the relationship between TCB and SCA (see Figure 2). In the structural equation model (Figure 2), all standardized path coefficients and significance levels are presented to provide clarity. The path from TCB to PSS is significant (β = 0.60, *p* < 0.001), as is the path from PSS to RS (β = 0.45, *p* < 0.001) and from RS to SCA (β = 0.34, *p* < 0.001). These results confirm the chain mediation model.

## 5 Discussion

This study investigates the relationships among socio-cultural adaptability (SCA), perceived social support (PSS), resilience (RS), and teachers’ caring behavior (TCB) experienced by international students studying in China. The findings validate several hypotheses through the construction of a chain mediation model that explores how TCB influences SCA in the context of online teaching, as well as the mediating roles of PSS and RS.

First, based on the cultural adaptation theory proposed by Ward et al., the study examined the SCA of international students who participated in online learning during the COVID-19 pandemic. The results help refine the objective factors influencing SCA and contribute to a deeper understanding of cultural adaptation theory. The study found no significant differences in SCA based on gender, student category, or learning style among international students in China, which differs from the findings of Fong (2020) but aligns with those of Kwak and Berry (2001). This discrepancy may be attributed to the Chinese government’s efforts to help international students integrate into Chinese life and better understand Chinese culture. Notably, “Overview of China” is a compulsory course required for all international students across Chinese universities, and passing this course is a prerequisite for graduation regardless of the student’s academic level. To ensure optimal learning outcomes, universities offer the course in bilingual formats and tailor the content according to the academic level of different students. Additionally, as part of the “Study Abroad in China” initiative, the Chinese government has prioritized enhancing the quality of teaching and international student satisfaction, which may also serve as an objective factor contributing to improved SCA.

Second, the results indicate that, even in online teaching environments, TCB is significantly and positively associated with SCA among international students in China, thereby confirming H1. This finding enriches the theoretical scope of cross-cultural adaptation, extends its application to online education, and underscores the crucial role teachers play in enhancing the SCA of international students. The study provides practical insights for fostering optimal teacher-student interaction. Furthermore, it affirms the positive psychological impact of educators and supports the effectiveness of online teaching in promoting teacher-student engagement, consistent with prior research (Osterman, 2023; Zhan et al., 2021). Such effective teacher-student communication plays a key role in supporting the SCA of cross-cultural learners (Fanari, 2025). Although online education creates physical distance between students and teachers, instructors are encouraged to adopt diverse interactive teaching strategies to deliver course content effectively, gather feedback, and maintain student engagement. This increased interaction aligns with the principles of TCB and is central to cross-cultural communication. Previous studies have confirmed that communication with locals enhances SCA (Ward and Kennedy, 1992), even in virtual contexts. Moreover, compared to face-to-face instruction, the frequency and diversity of teacher-student communication tools have significantly increased in online settings, facilitating deeper engagement and feedback.

Finally, the study further confirms that both PSS and RS play a partial chain mediating role between TCB and SCA. While PSS alone does not independently mediate the relationship between TCB and SCA, RS does function as an independent mediator. Therefore, PSS and RS jointly form a partial chain of mediation. As such, H2 is not supported, while H3 and H4 are validated. These findings enrich the understanding of the psychological mechanisms underlying SCA in international students, particularly in Eastern educational contexts, and add depth to cross-cultural adaptation theory. In addition to language proficiency and communication skills, the psychological experiences of cross-cultural learners play a pivotal role in SCA, offering a theoretical foundation for cross-cultural educational practices. The results also reveal that RS varies across student types and that gender differences exist in levels of PSS, consistent with previous research (Antonucci and Akiyama, 1987; Lyu C. 2024; Lyu Y. 2024; Winfield, 1991). Mediation analysis shows that RS significantly enhances SCA, suggesting that teachers’ caring behavior is linked to improved adaptability among international students in China, in line with prior studies (Liao et al., 2019; Romano et al., 2021; Tol et al., 2013). Although online communication, social interaction, and the provision of teaching resources (as aspects of TCB) offer social support for students (Cifuentes-Faura et al., 2021), this support was not significantly associated with improvements in SCA. These findings carry practical implications for fostering SCA in international students and offer further insight into the limited role of PSS in online contexts, supplementing previous findings (Burns et al., 2018; Chavajay, 2013; Jyoti and Kour, 2017). While the study confirms the feasibility of enhancing SCA through online instruction, it also emphasizes the need to strengthen students’ RS, as social support systems were not significantly correlated with SCA in an online learning context. For prospective international students preparing to study in China, early-stage training via online platforms could be beneficial in enhancing their SCA.

One notable limitation of the present study lies in the lack of measurement invariance testing across cultural subgroups. While all international students were treated as a single sample, differences in cultural interpretations of key constructs may exist. Due to the limited sample size, multi-group CFA by region or language of instruction could not be conducted. Future studies should incorporate such analyses to verify the consistency of factor structures and ensure cross-cultural comparability of findings.

The non-significant mediating effect of perceived social support (PSSS) in the relationship between teacher caring behavior (TCB) and socio-cultural adaptability (SCA) warrants further discussion. One possible explanation lies in cultural variation in how international students recognize or respond to perceived support. In some collectivist cultures, social support may be interpreted as implicit or indirect and not always consciously acknowledged. Furthermore, the widespread use of online and hybrid learning models during the pandemic may have reduced opportunities for students to engage in direct support-seeking behaviors from peers or teachers. These contextual and cultural factors may have weakened the role of perceived support as a psychological bridge between TCB and SCA.

## 6 Conclusion

The current study describes the relationship mechanism between TCB and SCA of international students studying in China and explores and details the mediating role of RS and PSS. The results identify RS as a partially independent mediator that can affect the relationship between TCB and SCA, and RS must mediate PSS to impact SCA. The current findings not only validate existing adaptation theories and increase our understanding of the relationships between TCB, PSS, RS, and SCA but also offer practical strategies for universities aiming to better support their international student populations. Meanwhile, these factors together could explain the relatively high SCA among international students in China compared to other contexts. Furthermore, the findings suggest that educators should pay more attention to the RS of international students because RS can not only directly promote international students’ SCA but also intervene in international students’ SCA by developing PSS, thereby improving RS and forming a virtuous circle.

This study provides theoretical contributions by extending the traditional cross-cultural adaptation framework into the context of online education. While most prior research focused on physical immersion in host cultures, our findings demonstrate that online teaching modes can also facilitate socio-cultural adaptation through mechanisms such as teacher caring behavior and resilience development. This reveals that the processes of adaptation are not limited to face-to-face interactions but can occur within virtual learning environments, which broadens the applicability of adaptation theory in a post-pandemic world.

From a practical standpoint, our findings offer actionable insights for universities aiming to improve the experience of international students. Specifically, institutions should emphasize the development of students’ resilience through both formal programs and informal support structures. Teacher training programs can also integrate modules on intercultural sensitivity and emotional support strategies to enhance the effectiveness of online instruction. Moreover, while perceived social support did not directly mediate socio-cultural adaptation in our model, it can still contribute to resilience and should be considered as a part of comprehensive support strategies.

It must be pointed out that online teaching is positively linked to international students’ SCA. The spread of the COVID-19 pandemic has been a severe blow to international higher education, but it is also a huge opportunity for online education. During the 3 years of the pandemic, online education has experienced unprecedented development, from refining online courses’ details to the check-in for classes, simulation experiments, online evaluations, exam systems, etc. And various communication apps have been used more frequently. The communication between teachers and students is not limited to small classrooms but extends to various parts of the world. Although separated by distance, the Internet helps teachers and students remove barriers, allowing them to have more communication channels and making it easier to open up and understand each other truly. This process facilitates students’ cross-cultural learning and is associated with higher levels of SCA. This extends the scope of cross-cultural adaptation research into hybrid education formats and provides timely insight post-pandemic. Therefore, developing online teaching is one way by which the internationalization of education in China can be achieved to effectively promote the improvement of the quality of education for international students.

## 7 Limitations and future directions

This study attempted to infer causality through path analysis, yet the cross-sectional design fundamentally limits any causal interpretation. Future longitudinal studies could further deepen the understanding of the impact mechanisms of SCA., such as measuring the effect of study time in China on SCA. Not only that, this study focuses on cross-cultural learning groups in China, but foreigners in China include too many other groups, so our study cannot cover all of them in the study subjects. Therefore, more in-depth exploration can be conducted from the following aspects in future research on SCA. Longitudinal research can be carried out further to deepen the understanding of the impact mechanism of SCA. Then, there is a need to expand the research object to other cross-cultural groups, provide a more comprehensive understanding of SCA, and propose feasible and targeted educational opinions. At the variable level, this study also fails to consider the duration of stay in China, Chinese language proficiency, cross-cultural experience, and other factors of the research subjects, which need to be further expanded in subsequent studies. A potential limitation of this study is the use of self-reported data from the same respondents, which may lead to shared-method variance. Although we conducted CFA comparisons to initially test for common-method bias, the risk of inflated correlations due to method effects cannot be completely ruled out. Future studies may benefit from incorporating multi-source or longitudinal data to mitigate this issue. Another limitation lies in the omission of potentially relevant covariates in the model. Factors such as students’ length of stay in China, previous cross-cultural experience, host language proficiency, and program level may influence their perceived social support, resilience, and socio-cultural adaptability. Future research should consider including these variables to better control for individual differences and improve the precision of model estimation.

## Data Availability

The original contributions presented in the study are included in the article/supplementary material, further inquiries can be directed to the corresponding authors.
