# Correction: How teachers' caring behavior impacts the socio-cultural adaptability of international students: a chain mediation model

**DOI:** 10.3389/fpsyg.2025.1730687

**Published:** 2025-11-11

**Authors:** Zilong Yin, Yuhao Zhao, Yuefang Wang

**Affiliations:** 1 School of Teacher Education, Jiangsu University, Zhenjiang, Jiangsu, China; 2 School of Psychology and Educational Sciences, Zaozhuang University, Zaozhuang, Shandong, China

**Keywords:** teachers' caring behavior, socio-cultural adaptability, perceived social support, resilience, international students

In the published article, the contributions of authors Zilong Yin and Yuhao Zhao were erroneously excluded.

The author list was incorrectly written as:

“Zilong Yin^1*^, Yuhao Zhao^1^ and Yuefang Wang^2*^”

The correct author list is:

“Zilong Yin^1*^^†^, Yuhao Zhao^1^^†^, and Yuefang Wang^2*^”

^†^These authors have contributed equally to this work

The original version of this article has been updated.

